# Birds Do It, Bees Do It, but Candida albicans Does It Differently

**DOI:** 10.1371/journal.pbio.0060121

**Published:** 2008-05-06

**Authors:** Richard Robinson

The yeast Candida albicans lives an unnoticed and mostly harmless life as a member of our gut flora. However, mainly in an immunocompromised host, it can proliferate and cause severe, life-threatening infections. Within this normally mild-mannered, single-celled fungus beats the heart of a reproductive adventurer. For while it appears to be incapable of meiosis and therefore true sex, it engages in an unusual and offbeat alternative—after it mates, its progeny randomly cast off chromosomes to restore the diploid number, or something close to it. In a new study, Anja Forche, Richard Bennett, and colleagues show that this process generates significant genetic diversity, which is further amplified by recombination between homologous chromosomes, using a protein that is elsewhere used exclusively in meiosis.

Meiosis is essential for sexual reproduction because it prevents the number of chromosomes from doubling every generation. In humans, 23 pairs of chromosomes (the “diploid” number) split at the start of meiosis to give each gamete cell 23 singletons (the “haploid” number). Union of sperm and egg restores the diploid number. Genetic diversity is an important consequence of meiosis—members of chromosomal pairs, called homologs, segregate randomly during meiosis, so that individual gametes contain a unique mix of paternally and maternally derived homologs. Diversity is increased when, before separating, homologs exchange segments, a process called recombination, or crossing over.

C. albicans, though, does none of that. Its diploid number is 16, and a reductional division to an 8-chromosome haploid has never been observed. Instead of going down, the number goes up—during conjugation, two different mating strains unite diploid cells to form tetraploid (32-chromosome) cells, which then reduce back toward the diploid number by chromosomal loss. This “parasexual” cycle has only recently been discovered, and its details and genetic consequences have not been well characterized.

**Figure:**
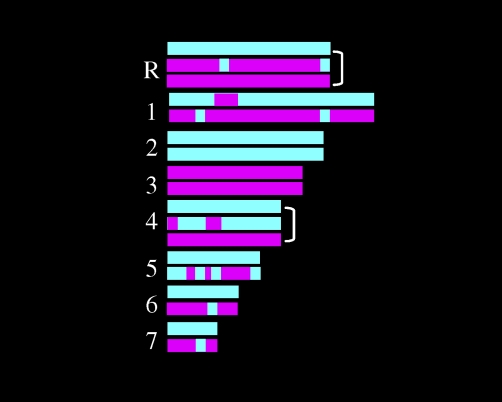
Configuration of the eight chromosomes of Candida albicans in a recombinant strain derived from the parasexual mating cycle.

The authors found that by manipulating the medium used to grow the yeast, they could induce stable tetraploids to undergo a reduction in their chromosome number. They isolated and analyzed the chromosome-reduced progeny, and showed that each strain was diploid, or nearly so. Colonies grown from these single cells displayed a wide range of phenotypes, from smooth (few filamentous cells) to wrinkled (many filamentous cells).

To examine the genetic diversity of the offspring further, they turned to single nucleotide polymorphism (SNP) profiling, combined with comparative genome hybridization (CGH). SNPs are short DNA sequences that differ between homologous chromosomes, thus allowing the identification of maternal versus paternal homologs in the offspring. CGH provides information on the copy number of each homolog present.

Their analysis showed that of 13 progeny strains, only three were true diploids, with exactly 16 chromosomes. The rest had three copies of at least one chromosome. Colonies from cells with extra chromosomes generally grew more slowly than those of true diploids. Viability did not require both maternal and paternal chromosomes in the same cell—two copies of either would suffice, suggesting that many chromosomes do not harbor lethal recessive alleles, as has been proposed for C. albicans.

Unexpectedly, the authors also found evidence that, in some strains, the remaining chromosomes had undergone homologous recombination, one of the hallmarks of meiosis. C. albicans is known to have genes that, in other organisms, function specifically in meiosis, but no role for them in C. albicans has been previously identified. The authors showed that one such protein, Spo11p, which in other species cleaves double-stranded DNA as a prelude to meiotic recombination, was expressed in mitotically growing C. albicans. When the researchers deleted the gene, tetraploid cells could still reduce their chromosome number, but did not undergo recombination. These results indicate that in C. albicans, Spo11 cleaves double-stranded DNA to bring about recombination but, instead of occurring during meiosis, this happens during chromosome loss in the parasexual cycle.

The authors propose that parasexuality may provide two advantages over the conventional meiotic sexual cycle, both tied to the yeast’s ability to survive within the mammalian host. First, the randomness of chromosome reduction and the high tolerance of the presence of three copies rather than two (or four) increases genetic variety, a potentially important feature for living in a dynamic environment such as the intestine. Second, the parasexual cycle avoids the production of spores, often the end result of fungal meioses. Spores are believed to be highly conspicuous to the immune system, which is exactly what such a low-profile resident of the gut wants to avoid.

